# 

**Published:** 1896-07

**Authors:** 


### CONTENTS—JULY, 1896.—VOL. XII., NO. 1.

ORIGINAL COMMUNICATIONS—

On the Proper Treatment of Consumption. C. H. Wilkinson, M. D., Galveston . . . . . 1

The Effect and Result of Sero-therapy in Tuberculosis. Paul Paquin, M. D., St. Louis . . . . . 8

Hypertrophy of the Pharyngeal Tonsil. H. L. Hilgartner, M. D., Austin . . . . . 18

SOCIETY NOTES—

Texas State Medical Association; Announcement of Officers of Sections for 1896-7 . . . . . 12

Texas State Medical Association; A Card to Members of Local Societies . . . . . 23

EDITORIAL—

Expediency vs. Principle . . . . . 25

Ethics of a Mixed Board of Medical Examiners; Letter from Prof. N. S. Davis, M. D . . . . . 29

Reminiscent—1877 vs. 1896 . . . . . 31

The Pretext, 35. Expediency, 36 . . . . . 35, 36

That Resolution . . . . . 36

Another Clincher . . . . . 38

Let Him That is Without Sin Cast the First Stone . . . . . 41

ABSTRACTS AND SELECTIONS—

Farewell Address to the Class of 1896. Marion-Sims Medical College. Prof. A. C. Bernays . . . . . 43

THE JOURNAL'S OWN PAGE—

Our 12th Voyage . . . . . 45

MEDICAL NEWS AND MISCELLANY—

Numerous paragraphs; Items of interest to the Doctor . . . . . 47

BOOK NOTICES—

Twentieth Century Practice of Medicine . . . . . 52

PUBLISHERS' NOTES . . . . . 53

New Advertisements, Medical and other colleges . . . . . 53

